# Energy Harvesting Under Dim-Light Condition With Dye-Sensitized and Perovskite Solar Cells

**DOI:** 10.3389/fchem.2019.00209

**Published:** 2019-04-09

**Authors:** Sean Sung-Yen Juang, Pei-Ying Lin, Yu-Chiung Lin, Yu-Sheng Chen, Po-Shen Shen, Yu-Ling Guo, Yu-Chun Wu, Peter Chen

**Affiliations:** ^1^Department of Photonics, National Cheng Kung University, Tainan, Taiwan; ^2^Department of Resources Engineering, National Cheng Kung University, Tainan, Taiwan; ^3^Center for Micro/Nano Science and Technology, Tainan, Taiwan; ^4^Hierarchical Green-Energy Materials Research Center, National Cheng Kung University, Tainan, Taiwan

**Keywords:** dye-sensitized solar cell, perovskite solar cell, dim-light application, cobalt-based electrolyte, triple cations perovskite

## Abstract

We demonstrate highly efficient energy harvesting devices for dim-light application under 200 lux irradiation using dye-sensitized solar cells (DSCs) and perovskite solar cells (PSCs). The high-efficiency DSCs are composed of cobalt-based redox mediators in 3-methoxypropionitrile (MPN) solvent with MK-2 sensitizer. With the introduction of under layer treatment and fine-tuning of compositions in cobalt-based electrolyte, the power conversion efficiency of cobalt-based DSCs achieves 16.0% under 200 lux illumination. That outperforms the best device using the conventional iodine-based electrolyte illuminated with the same light intensity. Especially, cobalt-based electrolyte system exhibits a higher open circuit voltage than iodine-based electrolyte counterpart. We also investigate perovskite solar cells under dim-light condition. PSCs show higher open circuit voltage and short circuit current density than DSCs with efficiency up to 23.4%. In this work, our results demonstrate the promising potential of DSCs and PSCs in the dim-light applications.

## Introduction

Recently, the development of energy harvesting devices for Internet of Things (IOTs) application is considered an important aspect to realize self-sustained operation. Among the various choices of available energy from the environment, light is one of the most abundant with relatively high energy density. However, most of the solar cells are not perfect energy harvesters under low light conditions due to the weakness of being a minority carrier device.

Dye-sensitized Solar Cell (DSC), one of the emerging photovoltaic (PV) techniques, has several advantages such as being colorful, flexible, and having a relatively low fabrication cost. Therefore, it is regarded as the next generation PV technology to compete with conventional inorganic solar cells in the aforementioned unique aspects. The traditional dye-sensitized solar cells (DSCs) are composed of dye-sensitized mesoporous TiO_2_ film, redox electrolyte and platinized counter electrode, which receive significant advancement by O'Regan and Gratzel (1991). Recently, with optimization (Ito et al., 2008; Yella et al., 2011; Tsao et al., 2012; Feldt et al., 2014; Kakiage et al., 2014; Mathew et al., 2014) of TiO_2_ film, light absorbers, electrolytes, and counter electrodes, DSC has achieved 14% recorded efficiency (Kakiage et al., 2015). The other rising photovoltaic devices, perovskite solar cells, receive tremendous attention due to their high efficiency with low cost fabrication processes (Zhao et al., 2017). Power conversion efficiency (PCE) under AM 1.5G illumination has achieved more than 22% (Saliba et al., 2016a). More recently, studies of their performances under low light are emerging since encouraging pioneering work done by Chen et al. They demonstrated inverted-type PSCs with the efficiency over 20% under dim-light condition (Chen et al., 2015).

As the age of the Internet of Things (IOT) emerges, the applications of portable electronics with self-sustainable energy harvesting abilities become the focus of research. Such devices prevent the use of a battery and avoid environmental concerns at disposal. Therefore, energy harvesting under indoor light sources such as houses, stores, factories, and hospitals is becoming essential. DSCs perform extremely well under low light conditions, better than conventional silicon solar cells, and their unique advantages are beneficial for utilization in such scenarios (Sridhar, 2011; Eliasson et al., 2012).

In the past, the I^−^/${\text{I}}_{3}^{-}$ redox mediator was chosen as the electrolyte for high-performing DSCs due to several advantageous characteristics such as fast dye regeneration and slow charge recombination (Boschloo and Hagfeldt, 2009). The electrolyte compositions have significant influence on device performance. Bella et al. reported that the different additive and salt concentrations in the electrolyte changed the photovoltaic properties and influenced device efficiency (Bella et al., 2014). However, some properties of I^−^/${\text{I}}_{3}^{-}$ redox mediators in the electrolyte such as partial light absorption and relatively negative redox potential could limit the performance of DSCs under low light operation. Lan et al. reported that the DSCs with iodine-based electrolytes performed well under low light condition as the triiodide concentration is reduced in iodine-based electrolytes (Lan et al., 2012). Cobalt complexes as redox mediators in the electrolyte is an alternative candidate for dim-light illumination for its advantages of less parasitic optical absorption and energy redundancy required for charge separation (Hamann, 2012).

In this study, we investigate the use of [Co(bpy)_3_]^3+/2+^ (bpy = 2,2′-bipyridine) redox mediators in 3-methoxypropionitrile (MPN) solvent with MK-2 dye under dim-light (200 lux) condition. The effects of redox concentration and electrolyte additives are scrutinized. Under the optimized condition, the photoelectric conversion efficiency under 200 lux achieved 16% with an aperture area of 0.09 cm^2^, which outperformed the best device using the conventional I^−^/${\text{I}}_{3}^{-}$ redox mediators with Z907 dye illuminated with the same light intensity.

## Materials and Methods

### Preparation of Materials, Electrolytes, and Dyes

The [Co(bpy)_3_](PF_6_)_2_/[Co(bpy)_3_](PF_6_)_3_ compounds were synthesized by following the procedure reported in the previous literature (Klahr and Hamann, 2009). The iodine-based electrolyte was prepared with 0.8 M 1,3-dimethylimidazolium iodide (DMII), 0.5 M 4-tert-butylpyridine (TBP), 0.01 M *I*^2^ and 0.1 M guanidinium thiocyanate (GuSCN) in 3-methoxypropionitrile (MPN) solvent. The cobalt-based electrolytes was composed of lithium-ion free or 0.1 M lithium perchlorate (LiClO_4_), 0.5–1.0 M TBP, 0.05–0.25 M [Co(bpy)_3_](PF_6_)_2_, and 0.015–0.035 M [Co(bpy)_3_](PF_6_)_3_ in MPN solvent. The 0.2 mM MK-2 dye was dissolved in the mixture of acetonitrile and toluene with volume ratio 1:1; the 0.3 mM Z907 dye was dissolved in the mixture of acetonitrile and tert-butyl alcohol with volume ratio 1:1. Chenodeoxycholic acid (CDCA) was added in all the dye solution (0.2 mM for MK-2 dye, 0.3 mM for Z907 dye).

### Fabrication of Home-Synthesized TiO_2_ Paste

The sol-gel TiO_2_ colloidal solution was prepared via microwave-assisted solvothermal method (Shen et al., 2014). Titanium isopropoxide was dissolved in isopropanol (IPA) with a concentration of 0.55 M as a precursor. Deionized water was added dropwise into the solutions in the molar ratio of [H_2_O]/[Ti] at 3 to induce hydrolysis and condensation reactions. The mixtures were magnetically stirred for 30 min and subsequently transferred into a Pyrex tube with 60% of volume filling ratio. The solvothermal reaction was performed in a microwave reactor (Discover Labmate, CEM). The temperature of this reaction was controlled at 220°C for 30 min. The as-obtained TiO_2_ colloidal solutions were used directly for the paste preparation. Ultrasonic horn was applied to the colloidal solutions for 300 s to improve the dispersion of crystallites in the solvent. Terpineol and ethyl cellulose were added together into the solvent and stirred at room temperature until terpineol and ethyl cellulose were completely dissolved in the solvent. The desired viscous paste was obtained by removing the solvent using a rotary evaporator at 35°C.

### Fabrication of Dye-Sensitized Solar Cells and Perovskite Solar Cells

The fluorine-doped tin oxide (FTO) glass was cleaned with detergent, deionized water, acetone, and ethanol by ultrasonic bath for 15 min, respectively. The TiO_2_ under layer was deposited onto FTO substrates by spray pyrolysis using 0.92 M titanium diisopropoxide bis(acetylacetonate) precursor solution diluted in ethanol at 500°C. The photoanodes were derived from two different TiO_2_ nanoparticles: one is the commercial paste (18 NRT, Dyesol, for iodine-based DSCs) and the other is home-synthesized TiO_2_ paste (30 nm for cobalt-based DSCs). The mesoporous TiO_2_ layers were screen-printed on the under layer with thickness of 4 μm and subsequently covered by 4 μm scattering layer (PST400, JGC). After sintering, a 40 mM TiCl_4_ treatment was employed and sintered at 450°C for 30 min. After cooling down to 80°C, the photoanodes were immersed in MK-2 dye solution for 8 h (cobalt-based) and Z907 dye for 6 h (iodine-based). Besides, for iodine-based photoanodes, we also immersed photoanodes in diisooctylphosphinic acid (DSPA) 10 mM solution for 15 min after dye uptake. The Pt-coated counter electrode was fabricated by dropping the H_2_PtCl_6_ solution on the FTO substrate and then sintered at 450°C for 30 min. Sandwich DSCs were sealed with photoanode and Pt-coated counter electrode by using the surlyn spacer (30 μm) at 130°C, and the iodine-based or cobalt-based electrolyte was injected into the hole which was drilled from the counter electrode. Finally, the hole was sealed by using thin glass with surlyn at 230°C. The cesium-containing triple cation perovskite solar cells were fabricated by following the procedure reported in the previous literature (Saliba et al., 2016b). The mixed perovskite precursor solutions contain 1 M FAI, 1.1 M PbI_2_, 0.2 M MABr, and 0.2 M PbBr_2_ dissolved in anhydrous DMF:DMSO with 4:1 (v/v) ratio. Then, the precursor was added to CsI solution which dissolved as a 1.5 M stock solution in DMSO. We chose spiro-OMeTAD as the hole-transporting material. The spiro-OMeTAD was doped with Li-TFSI and 4-tert-butylpyridine in chlorobenzene.

### Characterization

The dye absorption was measured by UV–vis spectrometer (U-4100, Hitachi). The J–V characteristics were measured under 200 lux illumination (65 μW/cm^2^) by using fluorescent T5 lamp (Philips T5 essential fluorescent tube) inside the black box and keithley source meter. The 200 lux spectrum was measured by the spectrometer (S-2440, Soma optics). Cyclic voltammetry (CV) and impedance measurements were measured by Autolab (PGSTAT30). Impedance measurements were conducted in the dark. The frequency was swept from 10 KHz to 0.1 Hz using an AC amplitude of 10 mV. Resistances and capacitances were analyzed using the software NOVA. The active areas of dye-solar cells and perovskite solar cells (PSCs) were defined with a mask by 0.09 and 0.20 cm^2^, respectively.

## Results and Discussion

**Figure 1:**
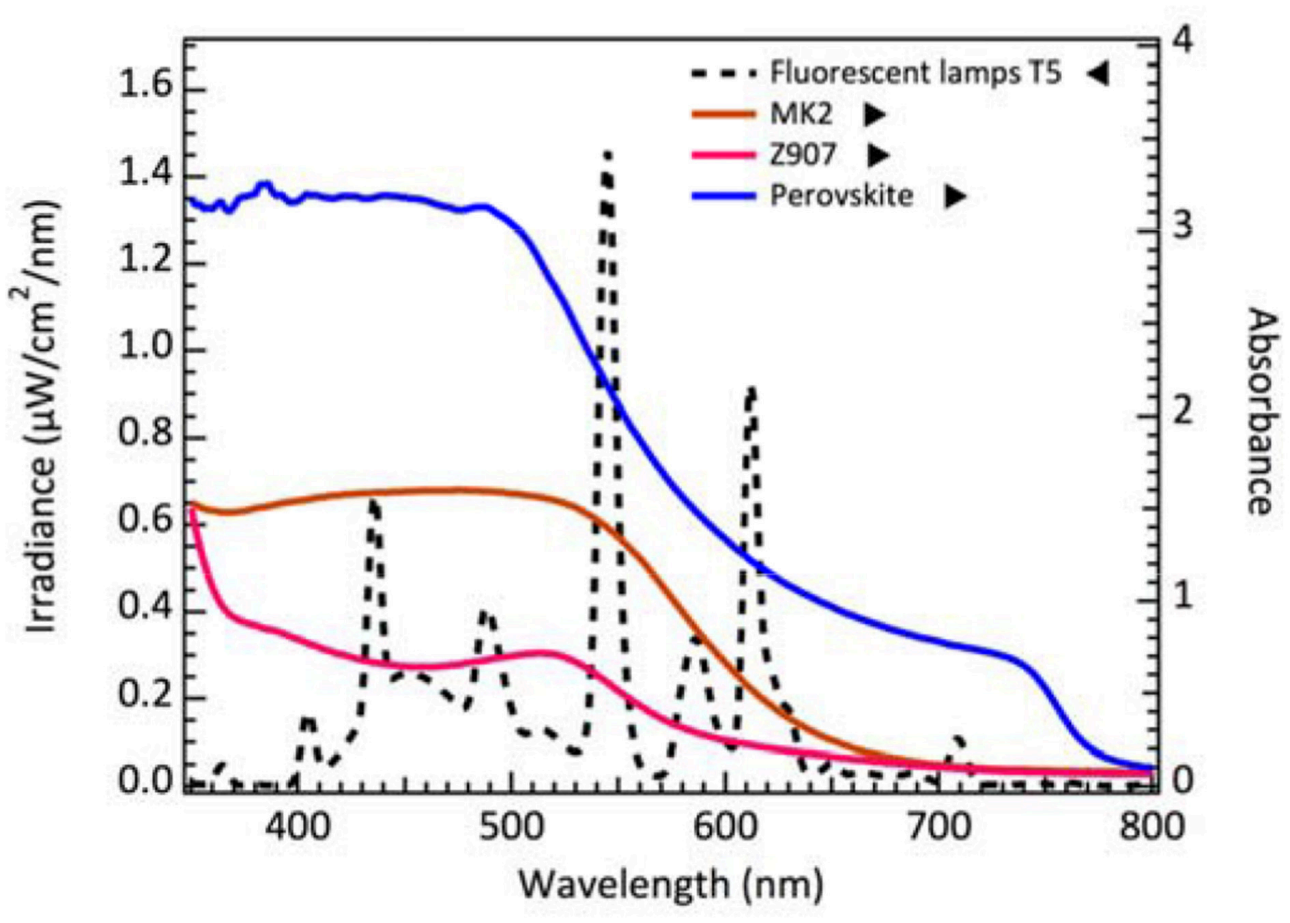
The spectrum of fluorescent lamp T5 irradiation and the absorption spectra of MK-2, Z907 dye on 2 μm thick TiO_2_ film and perovskite deposited on the mesoporous TiO_2_.

For dim-light condition measurements, we use T5 fluorescent lamp as the light source. The spectrum of fluorescent lamp T5 irradiation and the absorption spectra of MK-2, Z907 dye and perovskite are shown in Figure 1. From point of view of light harvesting, dyes with high extinction coefficient and a narrow absorption band below visible range might be better choices for the dim-light application. Omata et al. reported that the ruthenium dyes were not suitable in cobalt-based electrolyte system. They attributed the effects to the extra recombination channel (Omata et al., 2015) which resulted from the interaction of the Co redox with the Ru dye. Therefore, we select an organic dye MK-2, whose absorption spectrum overlaps ideally with the light source for cobalt-based electrolyte system.

### Effect of Blocking Layer Under Dim-Light Illumination

**Figure 2:**
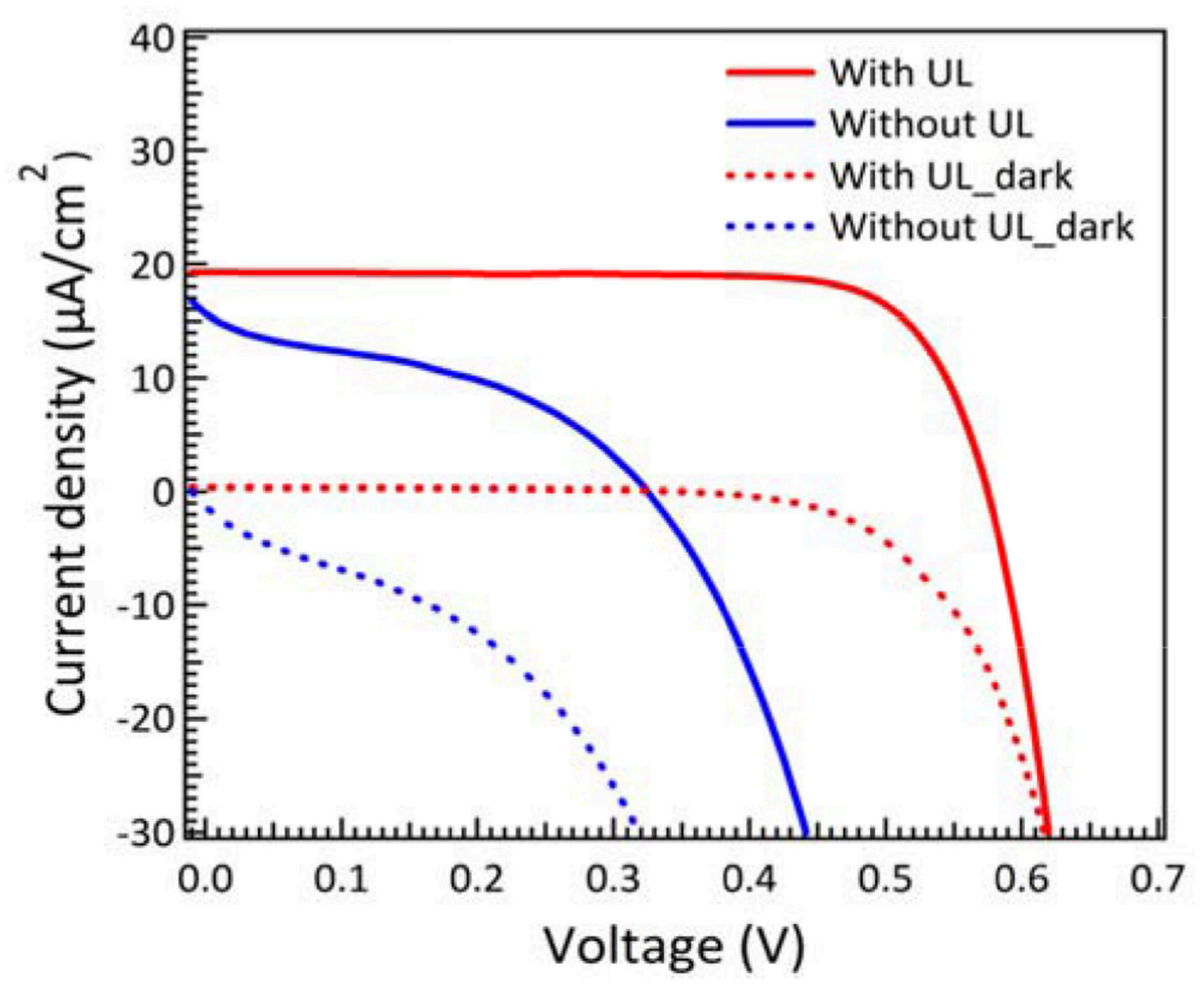
J–V characteristic curves of DSCs with and without blocking layer measured under 200 lux illumination. Electrolyte contains 0.1 M LiClO_4_, 0.2 M TBP, 0.33 M Co^2+^, and 0.05 M Co^3+^ complex in MPN.

Unlike the situation for high intensity illumination, the photon flux is very low in dim-light condition. In other words, light-induced electron transfer becomes much less than high intensity illumination in solar cells. As the result, the Jsc values are close to dark current density under low light condition. In order to reduce charge recombination, we introduce the under layer treatment as blocking layer to effectively prevent the charge recombination between the interfaces of FTO and electrolyte (Burke et al., 2008). The J-V characteristic curves of DSCs with and without under layer treatment are displayed in Figure 2. It is obvious that DSCs without under layer show a severely shunting curve and result in a much lower open-circuit voltage (*V*_*OC*_). In contrast, the DSCs with under layer show higher fill factor (FF) and *V*_*OC*_. The photovoltaic parameters for DSCs with and without under layer treatment measured under 200 lux condition are summarized in Table S1 for comparison. The results for higher *V*_*OC*_, *J*_*SC*_, and FF are attributed to the effective suppression of dark current and reduction of interfacial charge recombination. The efficiency for DSCs with under layer was almost 4 times higher than that without under layer under dim-light illumination.

### Effect of Compositions in Cobalt-Based Electrolyte Under Dim-Light Illumination

The DSCs are composed of a dye, anchored onto the surface of a wide-bandgap semiconductor, in contact with an electrolyte and closed by a counter electrode schematized in Figure S1. Open-circuit voltage (*V*_*OC*_) is defined as the difference between the quasi-Fermi energy level, *E*_*F*_ of the semiconductor and the potential of the redox system (Equation 1):

(1) $$V_{oc}=E_{F}-E_{Co3+/Co2+}$$

**Figure 3:**
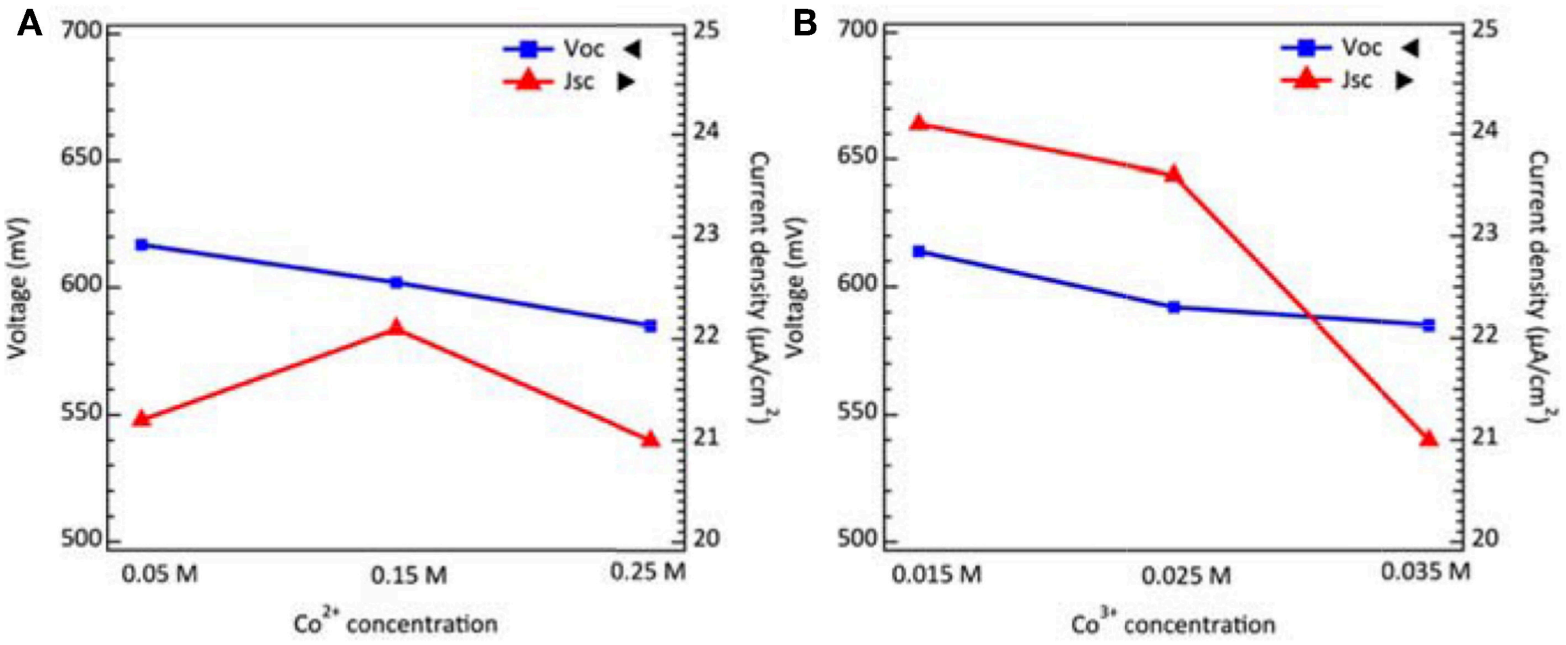
The voltage and current density of DSCs as a function of **(A)** Co^2+^ concentrations, and **(B)** Co^3+^ concentrations under 200 lux illumination.

For dim light condition, the composition in the electrolyte should be optimized for extreme low carrier concentration upon working condition. Bella et al. suggested the fallowing equation to study the effect of electrolyte concentration on device performance. First, we can consider the effect of the redox couple concentration as described by the Nernst equation (Bella et al., 2014):

(2) $$E=E_{0}-\frac{RT}{nF}ln\frac{\left[red\right]}{\left[ox\right]}$$

where *E*_0_ is the standard potential, *R* is the gas constant, *T* is the absolute temperature, *n* is the electron number, *F* is the Faraday constant, [*red*] and [*ox*] are the reduced and oxidized species concentration. As the concentration of reduced spices increase, the redox potential may shift to more negative values. From Equation (2), it can be inferred that the *V*_*OC*_ decreases while the Co^2+^ concentration increases as shown in Figure 3A with their photovoltaic parameters summarized in Table S2. Secondly, we kept the concentration of additives, salts, and Co^2+^ constant and varied the Co^3+^ concentration. The relation between Co^3+^ concentration including other dominant factors and *V*_*OC*_ can be interpreted by the following equation (Bella et al., 2014):

(3) $$V_{oc}=\frac{k_{B}T}{e}ln(\frac{J_{inj}}{k_{rec}n_{c,o}(Co^{3+})})$$

where *k*_*B*_ is the Boltzmann constant, *e* is the elementary charge, *J*_*inj*_ is the flux of injected electrons, *k*_*rec*_ is the recombination rate constant of Co^3+^ reduction and *n*_*c,o*_ is the collection of electron density on conduction band in the dark condition. The experimental results, well-supported by Equation (3), predict that the *V*_*OC*_ decreases while the Co^3+^ concentration increases as shown in Figure 3B with photovoltaic parameters summarized in Table S3. The concentration of oxidized species shall be reduced in dim-light conditions. To examine the limiting current under low concentration of redox mediator, we conduct the cyclic voltammetry (CV) analysis for dummy cells fabricating by different Co^2+^ and Co^3+^ concentrations. The results in Figures S2A,B indicate that the limit current is sufficient to carry the photocurrent under 200 lux conditions. Thus, the use of a low concentration redox couple in electrolyte mainly gains the voltage through thermodynamic and kinetic control without the compromise of charge transport.

**Figure 4:**
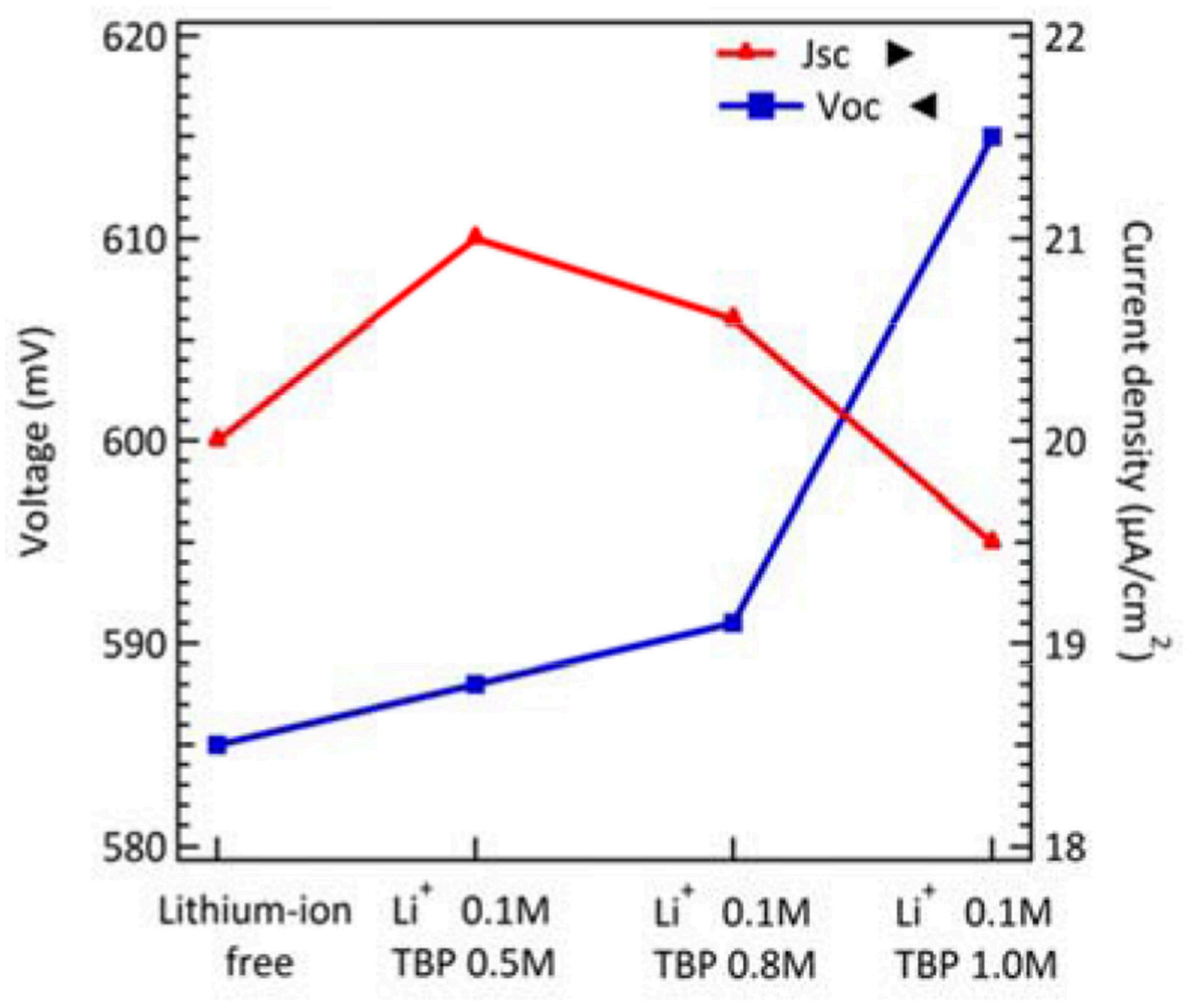
The voltage and current density of DSCs as a function of Li^+^ and TBP concentrations under 200 lux illumination.

**Figure 5:**
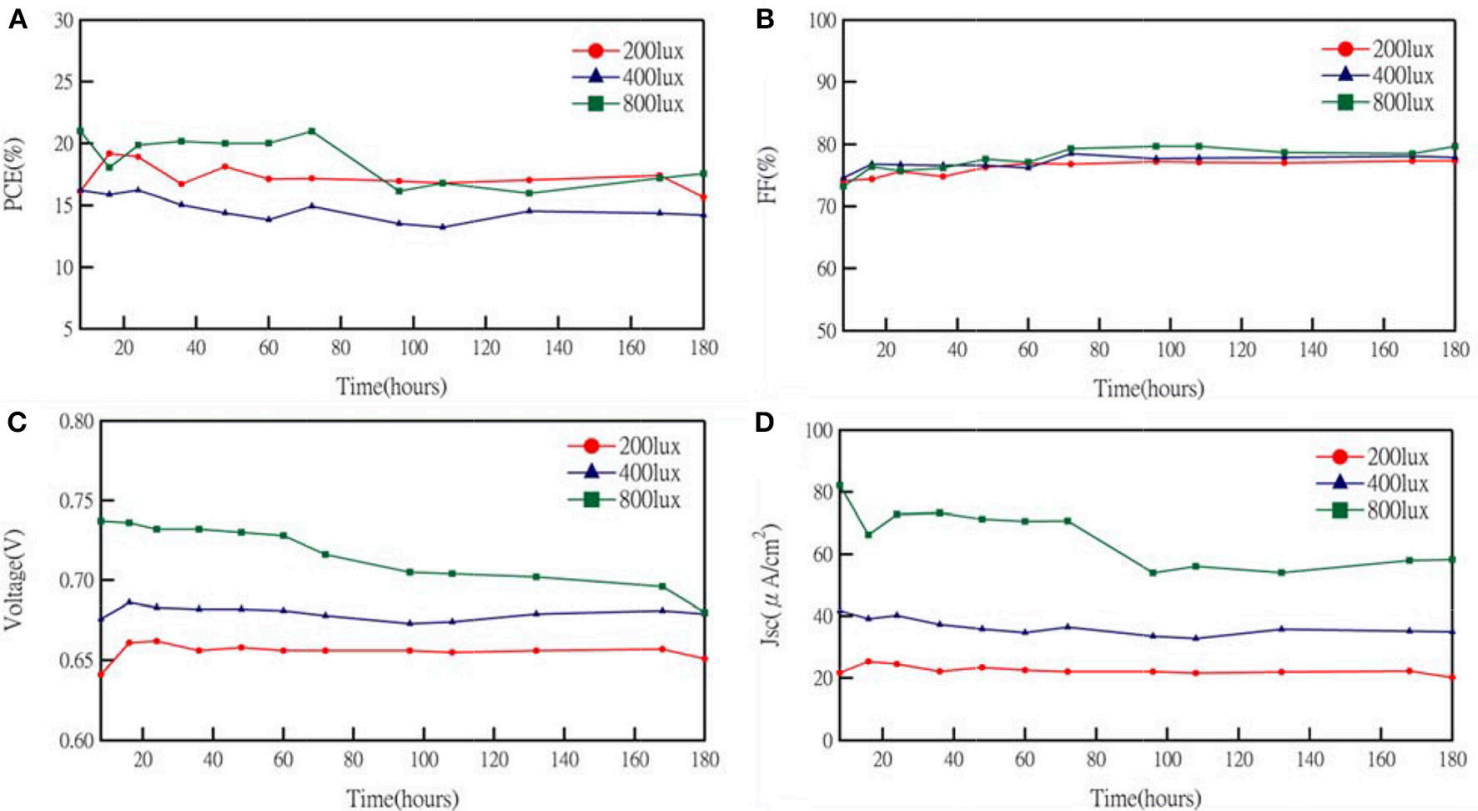
J–V characteristics of an cobalts-based device under light intensity illumination as a function of aging time: **(A)** PCE, **(B)** FF, **(C)** *V*_*OC*_, and **(D)** *J*_*SC*_.

Additives and salt also influenced the performance of DSCs. Koh et al. demonstrated the effect of TBP on the performance of cobalt-based DSCs. The adsorption of TBP onto the TiO_2_ surface can effectively reduce interfacial recombination. Although increasing TBP contents would cause a negative shift of the conduction band edge of TiO_2_ to obtain higher photovoltage, the short-circuit current decreased because the barrier for charge injection increased. Meanwhile, a cobalt-complex electrolyte would become more viscous, leading to the mass transport limitations (Koh et al., 2013) when TBP concentration increased. Besides, the influence of cation in cobalt-based electrolyte was demonstrated by Gao and co-workers. Li^+^ salt, a common electrolyte ingredient, is considered to shift the TiO_2_ conduction band more positively and to create random trap-state distribution to the metal-oxide surface. It is obvious that electron life time becomes shorter and dye degradation is accelerated in Li^+^-containing cells. In dim-light conditions, the results for the effect of TBP concentration are similar to previous reported literature (Gao et al., 2015). The increasing *V*_*OC*_ and decreasing *J*_*SC*_ followed with higher tBP concentration as shown in Figure 4 and their photovoltaic parameters are summarized in Table S4. However, the variation in TBP concentration seems not to affect the efficiency of DSCs much under dim-light conditions. On the other hand, the resulting *V*_*OC*_ and *J*_*SC*_ as a function of the concentration of Li^+^ cation under 200 lux condition are shown in Figure 4 with photovoltaic parameters summarized in Table S4. For lithium-ion free cells, it may have a charge transfer problem because the FF becomes lower under 200 lux illumination. We further studied the stability of the cobalt-based DSC under intensities of 200, 400, and 800 lux, light soaking tested during exposure to 70% RH at room temperature for about 180 h (Figure 5). The efficiency graph (Figure 5A) shows a degradation of 4, 12, and 17% with respect to their initial PCE under 200, 400, 800 lux, respectively. Under 800 lux illumination, the loss of performance is mainly due to the significant decay of the *J*_*SC*_. Although there is a slight decay of device performance under 800 lux conditions, a huge boost of conversion efficiency under 200lux, after aging for 180 h still keeps 96% of the initial PCE, suggesting that cobalt-based DSC would be a potential candidate for future indoor application.

**Figure 6:**
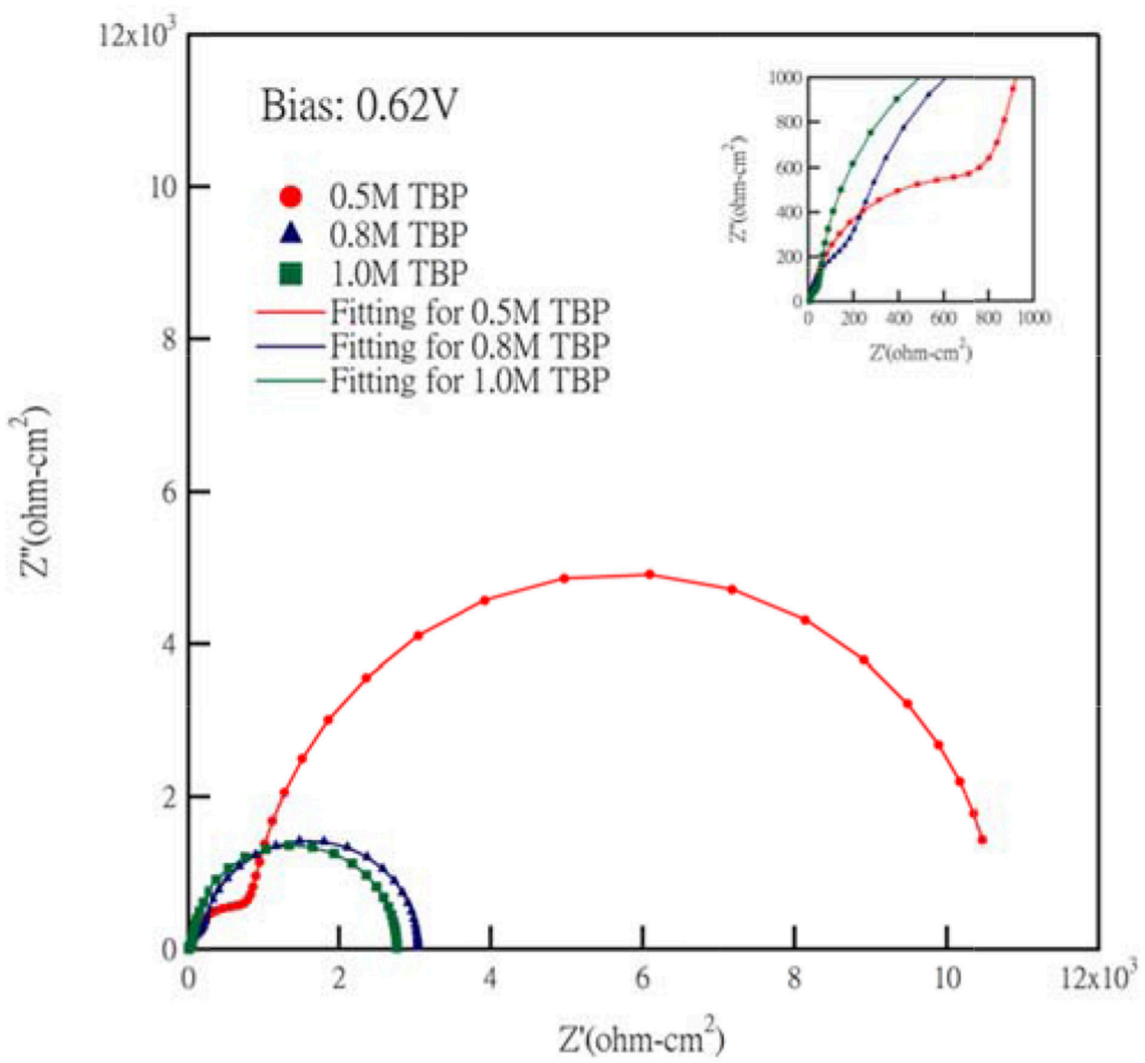
Nyquist plots and fitting curves of DSSCs with different TBP concentration.

Electrochemical impedance spectroscopy (EIS) measurement is widely used in the investigation of electrochemical characteristics and interfacial carrier transport properties (Bisquert and Fabregat-Santiago, 2010). In our study, EIS of different TBP concentrations at an apply bias 0.62 V are scanned, and the Nyquist plots including fitting curves are shown in Figure 6. By fitting curves of Nyquist plots, these impedance elements can be obtained and are summarized in Table 1. The equivalent circuit model for fitting the EIS spectra of cells is shown in Figure S3. Each semicircle represents a charge transfer process at different interfaces and the semicircle in the high frequency range corresponds to the charge transfer between counter electrode/electrolyte interface (R_CT_). The other semicircles in the low frequency range is attributed to the impedance related to electron recombination on the interface of TiO_2_/dye/electrolyte (R_TiO2_), and electrolyte diffusion (Z_D_), respectively. R_CT_ is a key parameter for a highly efficient DSSC. A large R_CT_ at the counter electrode implies that a charge transfer overpotential is required, which contributes to a voltage drop. (Lan et al., 2012) The value of R_CT_ in our experiments changed from 272 to 6,960 ohm-cm^2^ as we decreased the TBP concentration, which corresponds to the voltage drop caused by the charge transfer overpotential of about 30 mV. Another factor for the increase of *V*_*OC*_ as we increase the TBP concentration is that the TiO_2_ band edge can be shifted upward (negative in potential). The influence of TBP concentration on the I-V characteristics of Co-complex based DSCs has been identified previously in the literature. The lower current and higher voltage as we increase the TBP may be attributed to the barrier required for charge injection, as well as the increased viscosity of electrolyte, leading to the reduced *J*_*SC*_ (Koh et al., 2013).

**Table 1:** Photovoltaic parameters of DSCs made with iodine-based electrolyte and cobalt-based electrolyte and PSCs, respectively, under 200 lux illumination.

**Sample**	**V_**OC**_ (mV)**	**J_**SC**_ (μA/cm^**2**^)**	**FF**	**Output power (μW/cm^**2**^)**	**PCE (%)**
DSC-Iodine based	541 ± 69	17.8 ± 0.4	0.69 ± 0.03	6.7 ± 0.6	9.8 ± 0.9
	552	18.4	0.7	7.11	10.9
DSC-Cobalt based	638 ± 50	23.27 ± 0.6	0.67 ± 0.06	10 ± 0.8	15.36 ± 1.3
	641	23.36	0.73	10.8	16.7
PSC	775 ± 50	25.8 ± 0.5	0.63 ± 0.07	13 ± 2.5	21.7 ± 0.7
	797	26.4	0.72	15.2	23.4

### PSCs Under Dim-Light Illumination

**Figure 7:**
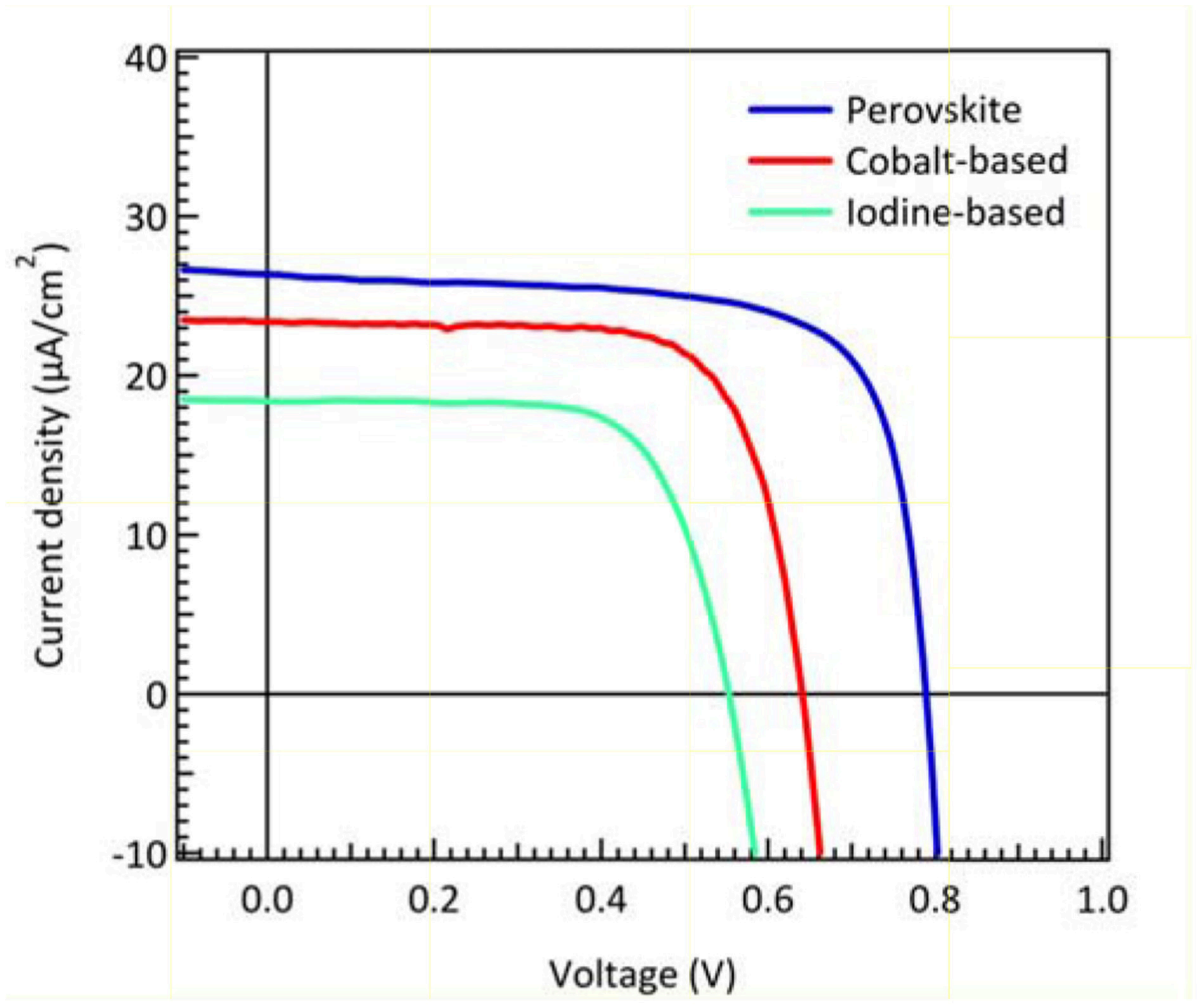
J–V characteristic curves of DSCs made with iodine-based electrolyte and cobalt-based electrolyte and PSCs, respectively, under 200 lux illumination.

Organometallic perovskite materials are direct band gap semiconductors with strong visible light absorption. Low trap-state density and long charge carrier diffusion lengths caused by high photocurrent in PSCs (Shi et al., 2015). Although a compound composed with organic-inorganic constituents, their high voltage response at high light intensity (AM 1.5G) implies minimum intrinsic loss. Such high voltage output approaches thermodynamic limit (Bi et al., 2016; Jacobsson et al., 2016; Saliba et al., 2016a). Therefore, PSCs are expected to get great performance in indoor applications. The light intensity dependence has been demonstrated in the previous report (Raifuku et al., 2016). The perovskite solar cells still maintained higher *V*_*OC*_ and *J*_*SC*_ than a-Si solar cells under low light illumination. Thus, it is very likely that perovskite materials could perform perfectly under dim-light conditions. Itaru et al. suggested that the planar structured PSCs showed better characteristics than mesoscopic structured ones under low light illumination (Raifuku et al., 2016). Chen et al. demonstrated the pioneering work of inverted-type PSCs with an efficiency of 23.9% under 200 lux condition (Chen et al., 2015). In this report, we fabricate the cesium-containing triple cation perovskite solar cells to compare with DSCs under 200 lux illumination. Cs^+^-doped perovskite was suggested to enhance optical and electrical parameters in PSCs (Saliba et al., 2016b). An efficiency of 23.4% was achieved under 200 lux condition, which is compatible with Chen's group PSCs performance. The *J-V* curves and photovoltaic parameters of best performing DSCs (made with iodine-based and cobalt-based electrolyte) and perovskite solar cells under dim-light condition were summarized in Figure 7 and Table 2. The results indicate that the efficiency of DSCs with cobalt-based electrolytes is higher than those with iodine-based electrolytes under dim-light conditions. Due to partial light absorption and relative negative redox potential of iodine-based electrolytes, the parameters of *V*_*OC*_ and *J*_*SC*_ are lower than those with cobalt-based electrolytes. Perovskite exhibits strong visible light absorption (shown in Figure 1) and high voltage response, providing even higher *V*_*OC*_ and *J*_*SC*_ than DSCs when worked in the dim-light illumination.

**Table 2:** EIS parameter of DSSC employed with different TBP concentration.

**Sample**	**R_**s**_(ohm-cm^**2**^)**	**R_**CT**_(ohm-cm^**2**^)**	**R_**TiO2**_(ohm-cm^**2**^)**
0.1 M Li${}_{-}^{+}$ + 0.5 M TBP	12	6,960	57,697
0.1 M Li${}_{-}^{+}$ + 0.8 M TBP	44	1,275	17,652
0.1 M Li${}_{-}^{+}$ + 1.0 M TBP	34	272	16,950

### Energy Harvesting Efficiency of DSCs and PSCs

Several previous reports of DSCs and PSCs in dim-light conditions (fluorescent lamp T5 as light source) are summarized in Table 3 for comparison. De Rossi et al. demonstrated iodine-based DSCs with N719 dye, and the efficiency achieved 12.6% under 200 lux illumination (De Rossi et al., 2015). Wang et al. demonstrated large-area (36.0 cm^2^) iodine-based DSCs, delivering PCEs of 3.11% with Z970 dye and 2.42% with AN-3 dye under 200 lux illumination (Wang et al., 2015). Liu and co-workers demonstrated DSCs of PCE 16.4% which was fabricated using iodine-based DSCs with porphyrin Y1A1 dye under 350 lux illumination (Liu et al., 2016). Because the absorption spectrum of porphyrin dye overlaps ideally with the LED light source, the efficiency could be higher under LED dim-light illumination. In this work, PCEs of 11.0 and 16.0% are demonstrated for DSCs of iodine-based electrolytes with Z907 dye and cobalt-based electrolyte with MK2 dye under 200 lux illumination. One advantageous property of cobalt complexes as redox mediators is its relatively reduced hole transfer overpotential which results in higher *V*_*OC*_. The results confirm that the efficiency of DSCs with cobalt-based electrolyte is relatively high among DSCs under dim-light conditions.

**Table 3:** List of photovoltaic parameters of previous reports (marked by black) and our current work (marked by red) performed under dim-light condition with T5 lamp.

**Sample**	**Active area**	**V_**OC**_ (mV)**	**J_**SC**_ (μA/cm^**2**^)**	**FF**	**PCE (%)**	**Lux intensity**	**References**
**DYE SENSITIZED SOLAR CELL (DSC)**							
DSC-iodine based (N719)	33.6 cm^2^	530	6.08	0.90	4.56	200 lux	De Rossi et al., 2015
DSC-iodine based (N719)	0.25 cm^2^	549	20.6	0.72	12.6	200 lux	De Rossi et al., 2015
DSC-iodine based (Z907)	36.0 cm^2^	363	10.0	0.54	2.42	200 lux	Wang et al., 2015
DSC-iodine based (AN-3)	36.0 cm^2^	362	8.0	0.54	2.42	200 lux	Wang et al., 2015
DSC-iodine based (Y1A1)	0.36 cm^2^	471	50.9	0.75	16.4	350 lux	Liu et al., 2016
DSC-iodine based (Z907)	0.09 cm^2^	552	18.4	0.70	11.0	200 lux	This work
DSC-cobalt based (MK-2)	0.09 cm^2^	641	23.36	0.73	16.7	200 lux	This work
**PEROVSKITE SOLAR CELL (PSC)**							
PSC (n-i-p heterojunction)	0.2 cm^2^	797	26.4	0.72	23.4	200 lux	This work
Inverted PSC (p-i-n heterojunction)	0.051 cm^2^	800	27.96	0.72	23.9	200 lux	Chen et al., 2015

We also calculate the theoretical limit current density from the dye and perovskite absorption spectrum integrating with the 200 lux spectrum as referred to Figure 1. The value of maximum current density is ~27 μA/cm^2^ for DSCs while ~28 μA/cm^2^ for perovskite solar cells. From the measured current density of cobalt-based and iodine-based DSCs, the current density of cobalt-based DSCs is close to the limit value. Consequently, we believe that reducing the potential loss is a key factor to achieve higher V_OC_ and further improve the performance in DSC for low light applications. Recently, Yasemin et al. demonstrated copper-based complex redox mediators for DSCs. Copper-based complex redox mediators are known for their more positive redox potential than cobalt-based redox mediators. DSCs will receive higher open-circuit voltage using these copper complexes as they can sufficiently regenerate the oxidized dye molecules with driving force potential as low as 0.1 V. In this result, the open-circuit voltage of DSCs can be achieved close to 1 V when using copper-based electrolyte with Y123 dye under AM 1.5G illumination (Saygili et al., 2016). Freitag et al. demonstrated even higher efficiency for copper-based DSCs under 200 lux condition. Their photovoltaic parameters are close to PSCs under low light illumination. With simultaneously immersed XY1 and D35 dyes, the output power of 15.6 μW/cm^2^ and efficiency of 25.5% were achieved under 200 lux conditions (Freitag et al., 2017) (Osram 930 warm-white fluorescent light tube). Therefore, there is still full potential for DSCs to improve performance under dim-light illumination in the near future.

## Conclusions

In summary, we demonstrate the strategies to optimize the efficiency of DSCs in dim-light application for the cobalt-based electrolyte system. Blocking layer effect, concentration of cobalt complexes (as redox mediators), additives and salt compositions investigated for their performances in DSCs under the dim-light condition. In this work, the efficiency of DSCs with cobalt-based electrolyte achieves 16%, which is greater than that with iodine-based electrolytes. We also compare DSCs with PSCs in dim-light condition. PSCs provide higher performance than DSCs both in voltage and current. By replacing iodine-based electrolyte with cobalt-based electrolyte in DSCs, we realize that reducing the potential loss for higher *V*_*OC*_ is the key to further improve the performance in DSCs under low light illumination. We believe the results revealed in our study are useful for developing further indoor applications in the future.

## Author Contributions

SS-YJ, P-YL, Y-CL, Y-SC, P-SS, Y-LG, Y-CW, and PC conceived and planned the experiments. SS-YJ, P-YL, Y-CL, Y-SC, and P-SS carried out the experiments and measurements. SS-YJ and P-YL derived the models and analyzed the data. SS-YJ, P-YL, Y-CL, Y-SC, and PC contributed to the interpretation of the results. SS-YJ and P-YL, took the lead in writing the manuscript. All authors provided critical feedback and helped shape the research, analysis and manuscript.

### Conflict of Interest Statement

The authors declare that the research was conducted in the absence of any commercial or financial relationships that could be construed as a potential conflict of interest.
