# Supplementary material for: Energy Harvesting Under Dim-Light Condition With Dye-Sensitized and Perovskite Solar Cells
Source: Front Chem. 2019 Apr 9;7:209. doi: 10.3389/fchem.2019.00209 (PMC6465951; doi:10.3389/fchem.2019.00209)

Supplementary Material

Energy Harvesting under Dim-Light condition with Dye-sensitized and Perovskite Solar Cells

Sean Sung-Yen Juang[1‡], Pei-Ying Lin[1‡], Yu-Chiung Lin[1], Yu-Sheng Chen[1], Po-Shen Shen[1], Pei-Ying Lin[1], Yu-Ling Guo[2], Yu-Chun Wu[2], and Peter Chen[*1,3,4]

[1] Department of Photonics, National Cheng Kung University, No.1, University Road, Tainan, 701, Taiwan

[2] Department of Resources Engineering, National Cheng Kung University, No.1, University Road, Tainan, 701, Taiwan

[3] Center for Micro/Nano Science and Technology (CMNST), No.1, University Road, Tainan, 701, Taiwan

[4] Hierarchical Green-Energy Materials (Hi-GEM) Research Center, National Cheng Kung University, No.1, University Road, Tainan, 701, Taiwan

‡ These authors contributed equally to this work.

*** Correspondence:**
Corresponding Author
petercyc@mail.ncku.edu.tw

Table S1. The comparison of photovoltaic parameters for DSCs with and without blocking under layer (UL) measured under 200 lux illumination. Electrolyte contains 0.1 M $LiClO_4$, 0.2 M TBP, 0.33 M Co^{2+} and 0.05 M Co^{3+} complex in MPN.

Sample	V_{OC} (mV)	J_{SC} ($\mu A/cm^2$)	FF	PCE (%)
Without UL	324	15.6	0.40	3.1
With UL	576	17.3	0.74	11.3

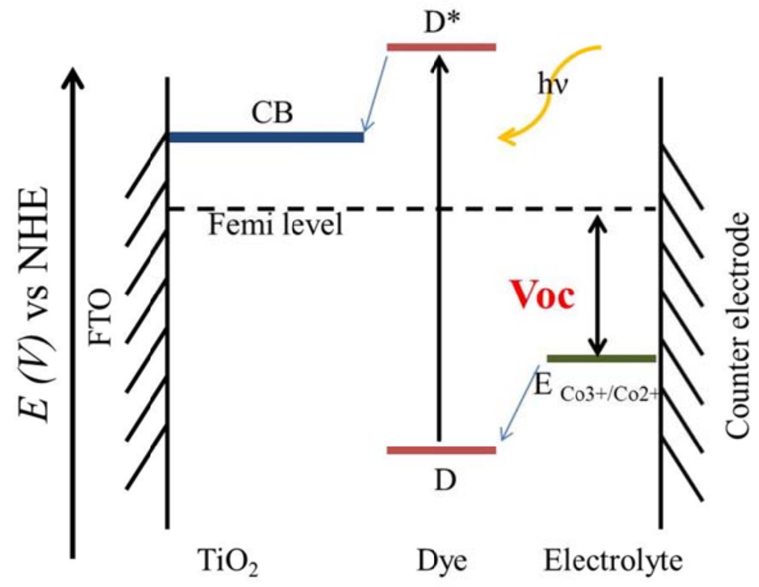

Figure S1. Working principles of a DSC. The open-circuit voltage of the cell is denoted V_{OC}.

Table S2. Photovoltaic parameters of cobalt-based DSCs made by different Co^{2+} concentrations under 200 lux illumination.

Sample	V_{OC} (mV)	J_{SC} ($\mu A/cm^2$)	FF	PCE (%)
0.05 M Co^{2+}	617	21.2	0.76	15.2
0.15 M Co^{2+}	602	22.1	0.78	15.8
0.25 M Co^{2+}	585	21.0	0.76	14.3

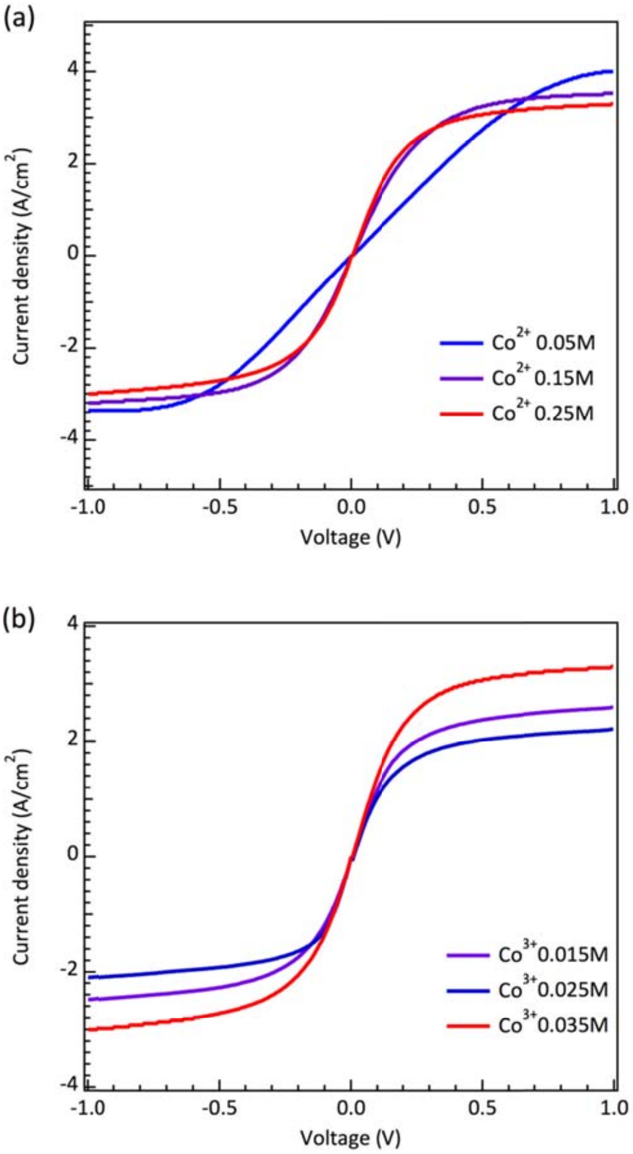

Figure S2. The cyclic voltammetry of dummy cells using different (a) Co^{2+} and (b) Co^{3+} concentration. The dummy cells were fabricated by two symmetry platinized electrodes, the area of surlyn is 0.36 cm^2.

Table S3. Photovoltaic parameters of cobalt-based DSCs made by different Co^{3+} concentrations under 200 lux illumination.

Sample	V_{OC} (mV)	J_{SC} (μA/cm^2)	FF	PCE (%)

0.015 M Co^{3+}	614	24.1	0.72	15.3
0.025 M Co^{3+}	592	23.6	0.75	15.8
0.035 M Co^{3+}	585	21.0	0.76	14.3

Table S4. Photovoltaic parameters of cobalt-based DSCs made by different Li^{+} and TBP concentrations under 200 lux illumination.

Sample	V_{OC} (mV)	J_{SC} ($\mu A/cm^2$)	FF	PCE (%)
Lithium-ion free TBP 0.5 M	588	20.0	0.61	10.5
LI^{+} 0.1 M TBP 0.5 M	585	21.0	0.76	14.3
LI^{+} 0.1 M TBP 0.8 M	591	20.6	0.76	14.2
LI^{+} 0.1 M TBP 1.0 M	615	19.5	0.77	14.2

Figure S3. Equivalent circuit model employed for fitting the EIS spectra of cells.

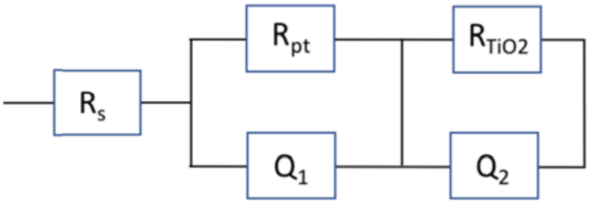

Supplement: Supplementary file 1 [file Data_Sheet_1.pdf]
